# Efficient and simple approach to *in vitro* culture of primary epithelial cancer cells

**DOI:** 10.1042/BSR20160208

**Published:** 2016-12-09

**Authors:** Karolina Janik, Marta Popeda, Joanna Peciak, Kamila Rosiak, Maciej Smolarz, Cezary Treda, Piotr Rieske, Ewelina Stoczynska-Fidelus, Magdalena Ksiazkiewicz

**Affiliations:** * Research and Development Unit, Celther Polska Ltd., Milionowa 23, 93-193 Lodz, Poland; † Department of Tumor Biology, Medical University of Lodz, Zeligowskiego 7/9, 90-752 Lodz, Poland

**Keywords:** breast cancer, extracellular matrix, primary cancer cell cultures, prostate cancer, Rho kinase (ROCK) inhibitor

## Abstract

Primary cancer cells constitute a favourable testing platform for *in vitro* research in oncology field as they reflect tumour state more accurately than the most commonly employed stable cell lines. Unfortunately, due to limited availability of material and difficulties with protocols validation, primary models are rarely implemented into laboratory practice.

We have compared protocols for primary cultures, differing in media components and plate coatings. In terms of culture establishment, application of Geltrex® coating demonstrated equal efficiency to feeder layer (83% compared with 72% successfully established breast and 80% compared with 80% prostate tumour specimens), yet it was substantially less complicated and easier to validate. Both Geltrex® coating and tissue-specific primary cell medium were permanently required to successfully maintain primary epithelial prostate cancer cells (PEPCs) in culture. In case of primary epithelial breast cancer cells (PEBCs), collagen I coating enabled to obtain comparable number of passages to Geltrex® coating (*P*=0.438). Commercial primary cell media demonstrated lower efficiency than tissue-specific ones (PEPCs–5 compared with 8 and PEBCs–6 compared with 9 passages). Interestingly, both analysed tumour types were unsusceptible to induction of culture lifespan extension when transduced with *SV40LT*, *BMI-1* or *hEST2* genes, commonly applied as potential immortalizing agents.

In conclusion, the approach based on extracellular matrix reconstitution and tissue-specific primary cell media is easy to validate and provides *in vitro* expansion sufficient for analytical purposes (approximately 8 passages). Therefore, it may facilitate implementation of hardly available experimental models for a variety of analyses.

## Abstract (precis)

Primary breast and prostate epithelial cancer cells may be efficiently cultured *in vitro* using simple and easily validatable approach–plates coated with a mixture of extracellular matrix components and tissue-specific primary cell medium.

## INTRODUCTION

Stable cancer cell lines have been considered valuable tools for analysis of cancer biology as well as applicable platforms for preclinical drug testing for many years [1,2]. Being relatively easy to culture (basic media requirements, simple culturing protocols) and having virtually limitless lifespan, these lines gained recognition among the majority of scientists. Since cancers are well known for high heterogeneity [3], stable cancer cell lines may be unable to adequately represent the complexity of these diseases [4,5]. Some tumour clones are more prone to *in vitro* maintenance, thus only a part of intratumoural molecular diversity is reflected in stabilized culture. Therefore, some tumours are under- or even unrepresented by *in vitro* cell lines. As an example, no stable cell line obtained from the primary prostate tumour site is available [6,7].

Primary cancer cultures currently begin to constitute the golden standard of *in vitro* model, hence their implementation into laboratory practice is highly recommended [8,9]. Limited time of culture prevents *in vitro* selection of single clones that are more prone to adaptation to artificial conditions and thus hinders stabilization of a homogeneous population. Therefore, primary cultures constitute a model that mimics *in vivo* tumour state better than stable cell lines. Such an approach, however, is associated with some technical hurdles–primary culture may be difficult not only to establish (e.g. due to poor quality of surgical material or cell culture shock associated with transition from *in vivo* to *in vitro* environment) but also to maintain for a sufficient number of passages to perform complex analyses (e.g. due to early onset of *in vitro* senescence [10,11]).

Many attempts have been made to overcome problems with epithelial primary cancer cultures, resulting in development of various strategies that employ special enriched media [12], feeder layer [13,14] or feeder layer and Rho kinase (ROCK) inhibitor-supplemented medium [15]. It has to be noted that feeder layer preparation is not only time-consuming, but also difficult to standardize. Mouse fibroblasts, most often employed as feeder cells, tend to transform or undergo senescence, thus they do not provide primary cells with adequate support [16]. Hence, usage of feeder layer coating is associated with a risk of inconsistencies in culture, affecting test outcomes. In the present study, we propose the protocol for primary epithelial prostate cancer (PC) and breast cancer (BC) cell cultures, involving application of extracellular matrix reconstitution along with tissue-specific primary cell medium. The proposed approach yields results comparable to feeder layer coating, is relatively simple and constitutes an easily validatable testing platform.

## MATERIALS AND METHODS

### Clinical material

Tumour samples were obtained from 18 patients diagnosed with BC and 25 diagnosed with PC, treated at the Polish Mother's Memorial Hospital Research Institute in Lodz or the Medical Centre in Pabianice and the Pirogow Hospital in Lodz respectively (Supplementary Table S1). All procedures were performed in accordance with the protocol approved by the Bioethical Committee of the Regional Medical Chamber in Lodz (Approval No. 3/12 of February 8, 2012). Patients signed informed consent form and their data were processed and stored according to the principles expressed in the Declaration of Helsinki. Each sample was collected by a surgeon in aseptic conditions and transferred into Hank's Balanced Salt Solution (HBSS; Gibco) supplemented with penicillin/streptomycin (1%; Biowest) and gentamycin (0.2%; Biowest). Tissue sections were handled no later than 3–4 h following their resection.

### Feeder layer preparation

NIH-3T3 cells (DSMZ) were cultured in DMEM HG (Gibco) supplemented with FBS (10%; Biowest), penicillin/streptomycin (1%) and gentamycin (0.2%). Cells were seeded onto attachment factor (Life Technologies) coated plates at density 1.8×10^5^/well. The following day, cells were inactivated with mitomycin C (2 h; 15 μl/ml; Cayman Chemical).

### Prostate cancer primary cultures

Prior to enzymatic dissociation prostate tumour samples were washed thrice in HBSS and mechanically disintegrated into pieces smaller than 1 mm^3^ using scalpels. Such tissue pieces were incubated (1.5 h, 37°C, on shaker) in a mixture of dispase (1 unit/ml; STEMCELL Technologies) and collagenase type IV (20 units/ml; Gibco) in DMEM:F12 (Biowest), followed by filtration through 100 μm filter. Subsequently, PC cells were seeded at density 0.5–1×10^5^/well (depending on tissue specimen size) onto Geltrex® (23 μg of protein per 1 cm^2^; Thermo Fisher Scientific), collagen I (15 μg of protein per 1 cm^2^; Thermo Fisher Scientific) or feeder layer coated six-well plates in either primary cell medium dedicated for PC cells (prostate cancer primary medium; PCPM), composing of DMEM:F12 supplemented with FBS (5%), penicillin/streptomycin (1%), gentamycin (0.2%), EGF (10 ng/ml; Sigma–Aldrich), adenine (20 μg/ml; Sigma–Aldrich), CHTX (8.4 ng/ml; Sigma–Aldrich), HEPES (15 mM; Thermo Scientific), insulin (5 μg/ml; PAN-Biotech), hydrocortisone (0.32 μg/ml; Sigma–Aldrich) and ROCK inhibitor (Y-27632; 10 μM; Merck Millipore) or commercial primary cell medium–PrEGM™ (Lonza). Cells were either further cultured and passaged using trypsin–EDTA (0.25%; Biowest) or frozen in FBS with DMSO (10%; Sigma–Aldrich) and–ROCK inhibitor (Y-27632; 10 μM).

### Breast cancer primary cultures

Prior to enzymatic dissociation breast tumour samples were washed thrice in HBSS and mechanically disintegrated into pieces smaller than 1 mm^3^ using scalpels. Such tissue pieces were incubated (16 h, 37°C, on shaker) in a mixture of collagenase/hyaluronidase (1×; STEMCELL Technologies) in DMEM:F12. Further disintegration was performed in two steps: *via* gentle pipetting in trypsin (0.25%) for 2 min and then in a solution of dispase (5 units/ml) and DNase I (0.05 mg/ml; STEMCELL Technologies) for 1 min. After each step, the enzymes were blocked with HBSS supplemented with 2% FBS and discarded with supernatant after centrifugation (150×***g***, 4 min). Subsequently, cells were seeded at density 0.5–1×10^5^/well (depending on tissue specimen size) onto Geltrex®, collagen I or feeder layer coated six-well plates in either primary cell medium dedicated for BC cells (Breast Cancer Primary Medium; BCPM), composing of DMEM:F12 supplemented with human serum (2%; Sigma–Aldrich), penicillin/streptomycin (1%), gentamycin (0.2%), EGF (10 ng/ml), adenine (20 μg/ml), CHTX (8.4 ng/ml), HEPES (15mM), insulin (5 μg/ml), hydrocortisone (0.32 μg/ml) and ROCK inhibitor (10 μM) or commercial primary cell medium–EpiCult™ (STEMCELL Technologies). Cells were either further cultured and passaged using trypsin–EDTA (0.25%) or frozen in FBS with DMSO (10%) and ROCK inhibitor (10 μM).

### Real-time quantitative reverse-transcription PCR (Real-time qRT-PCR)

RNA was isolated from frozen tumour samples (stored at −80°C) and corresponding primary cell cultures using AllPrep RNA/DNA Mini Kit (Qiagen) according to the manufacturer's protocol. Concentration of nucleic acids was measured spectrophotometrically (NanoPhotometer™; Implen) and 250 ng of total RNA was reverse-transcribed using QuantiTect Reverse Transcription Kit (Qiagen) according to the manufacturer's protocol. Real-time qRT-PCR was performed using TProfessional ThermoCycler (Biometra) and designed primers (Table 1) in biological and technical triplicates, as recently described [17]. In order to confirm the specificity of amplification signal, gene dissociation curve was considered in each case.

**Table 1:** Primer sequences

Real-time PCR	
*HPRT1*	For: 5′-TGAGGATTTGGAAAGGGTGT-3′
	Rev: 5′-GAGCACACAGAGG GCTACAA-3′
*TBP*	For: 5′-GAGCTGTGATGTGAAGTTTCC-3′
	Rev: 5′-TCTGGGTTTGATC ATTCTGTAG-3′
*MUC1/Y*	For: 5′-GCTGCTCCTCACAGTGCTTA-3′
	Rev: 5′-TGGGTAGCCGAAGTCTCCTT-3′
*MGB1*	For: 5′-TGCTGATGGTCCTCATGCTG-3′
	Rev: 5′-ACACTTGTGGATTGATTGTCTTGG-3′
*SNCG*	For: 5′-ACCAAGGAGGGGGTCATGTA-3′
	Rev: 5′-ACAGTGTTGACGCTGCTCAC-3′
*AMACR*	For: 5′-ATGGCTCTTTTTGACCGCAC-3′
	Rev: 5′-GGTGCTTCCCACAGACTCAA-3′
**Plasmid construction**	
*BMI-1*	For: 5′-GGGGACAAGTTTGTACAAAAAAGCAGCGTATGCATCGAACAACGAGAATCAAGATC-3′
	Rev: 5′-GGGGACCACTTTGTACAAGAAAGCTGGGTACCAGAAGAAGTTGCTGATGACCCATT-3′
*hEST2*	For: 5′-GGGGACAAGTTTGTACAAAAAAGCAGCGTATGCCGCGCGCTCCCCGCTGCCGAGCCGTG-3′
	Rev: 5′-GGGGACCACTTTGTACAAGAAAGCTGGGTGTCCAGGATGGTCTTGAAGTCTGAGGGCAG-3′
*SV40LT*	For: 5′-GGGGACAAGTTTGTACAAAAAAGCAGCGTATGGATAAAGTTTTAAACAGAGAGGAATCTTTGCAGC-3′
	Rev: 5′-GGGGACCACTTTGTACAAGAAAGCTGGGTTGTTTCAGGTTCAGGGGGAG-3′

*HPRT1*- and *TBP*-normalized relative gene expression levels of mammaglobin 1 (*MGB1*), mucin 1 isoform Y (*MUC1/Y*), synuclein gamma (*SNCG*) and α-methylacyl-CoA racemase (*AMACR*) in tested samples compared with control samples were calculated using the method described by Pfaffl et al. [18]. RNA isolated from MDA-MB-468 BC cell line (DSMZ) was used as a positive control for *MGB1, MUC1/Y* and *SNCG* mRNA expression in BC samples. Pooled cDNA from analysed prostate tissues showing average *MUC1/Y* and *AMACR* expression was used as positive control for expression of these genes. RNA isolated from normal cells–BJ human neonatal foreskin fibroblasts (ATCC) served as a negative control in these experiments, whereas no template control (NTC) reactions were used to exclude any PCR contaminations.

### Immunocytochemistry

Cells were fixed with ice-cold methanol, incubated with 2% donkey serum (1 h, room temperature; Sigma–Aldrich) to block non-specific binding, and subsequently, with the appropriate primary/secondary antibodies and DAPI (Sigma–Aldrich). Coverslips were mounted with mounting medium–N-propyl gallate (0.1 M; Sigma–Aldrich) in glycerol/PBS (9:1; Sigma–Aldrich and Biowest respectively) and analysed using Eclipse Ci fluo-rescent microscope (Nikon). The following antibodies were used: anti-pan-cytokeratin (pCK; 1:200; Santa Cruz Biotechnology), anti-MGB 1 (1:60; Santa Cruz Biotechnology), anti-CD90 (1:1000; Dianova), anti-mouse Alexa Fluor®594 and anti-rabbit Alexa Fluor®488 (both 1:500; Invitrogen).

### Multiplex ligation-dependent probe amplification

The multiplex ligation-dependent probe amplification (MLPA) reactions were performed as recently described [10] using commercially available probemixes (P173 and P105) and kits (MRC-Holland) following the manufacturer's recommendations. Fragments were separated by capillary electrophoresis using ABI 3130 Genetic Analyzer (Applied BioSystems). The comparative analyses were performed using Coffalyzer.Net (MRC-Holland). For each gene, the resultant ratio was calculated and interpreted as gain (above 1.3) or loss (below 0.7).

### Plasmids construction

RNA from NTERA-2 cell line (ATCC) was isolated using AllPrep DNA/RNA Mini Kit and reverse transcribed with QuantiTect Reverse Transcription Kit according to manufacturer's instructions. Q5® Hot Start High-Fidelity DNA Polymerase (NEB) was used to amplify protein coding cDNA sequences of SV40 large T antigen (*SV40LT*), *BMI-1* and *hEST2* genes with Gateway® specific primers (Table 1). PCR products were cloned into pENTR™/Zeo vector and subsequently transferred to pLV1/puro-DEST vector (generated as described recently [17]) using Gateway® Cloning Technology (Life Technologies) according to the manufacturer's protocol. At each step, sequences were confirmed with ABI 3130 Genetic Analyzer.

### Transduction with immortalizing genes

Lentiviruses carrying cDNA sequences of *SV40LT*, *BMI-1* and *hEST2* were prepared in HEK293 cell line (ATCC) using LENTI-Smart kit (InvivoGen) according to the manufacturer's recommendations. Primary cancer cells were transduced with either single gene or their combination (*SV40LT* with *BMI-1* or *hEST2*). Puromycin positive selection was applied 48 h post transduction (0.5 μg/ml; InvivoGen).

### Senescence-associated β-galactosidase staining

Senescence detection was performed using Histochemical Staining Kit (Sigma–Aldrich) according to the manufacturer's protocol. Briefly, cells were washed thrice with PBS and fixed with 4% paraformaldehyde (12 min). Subsequent washes with PBS (2×10 min) were followed by an incubation (overnight, 37°C, without CO_2_) with freshly prepared staining mixture (1 mg/ml X-gal, 5 mM potassium ferricyanide, 5 mM potassium ferrocyanide, 1× staining solution; X-gal pre-warmed at 37°C for 1 h). After the incubation, cells were washed thrice with PBS and analysed using light microscope (Opta-Tech).

### Statistical analysis

Statistical analyses were performed using RStudio software (RStudio) and GraphPad Prism Software (GraphPad Software). Two tailed *t*-test was employed for parametric results, whereas Wilcoxon test was used for non-parametric results, both with a value of *P*  <0.05 considered statistically significant.

## RESULTS

### Primary epithelial cancer cultures can be efficiently established *in vitro* on Geltrex®-coated plates

**Figure 1:**
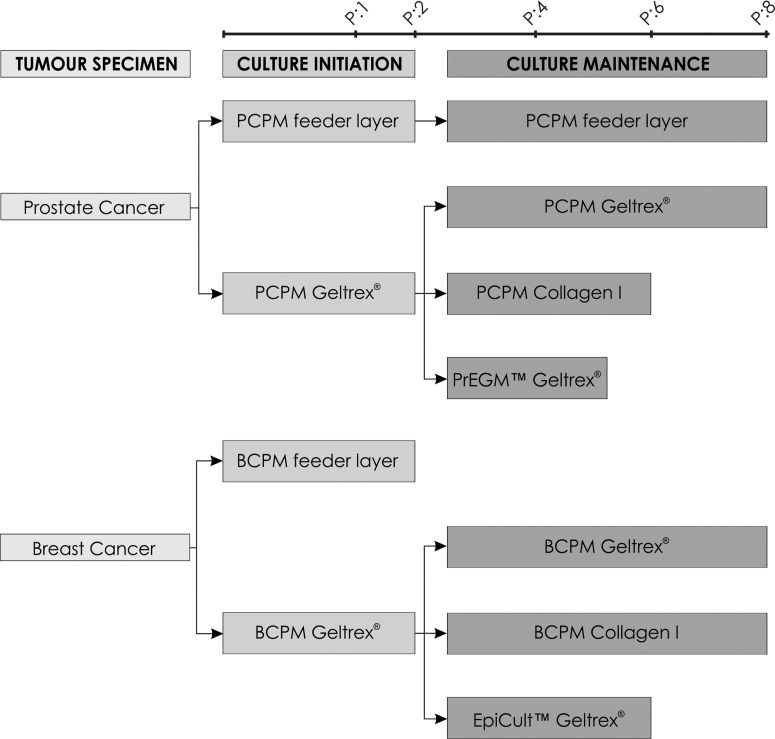
Schematic experiment workflow–various attempts to culture primary epithelial breast and prostate cancer cells.

**Figure 2:**
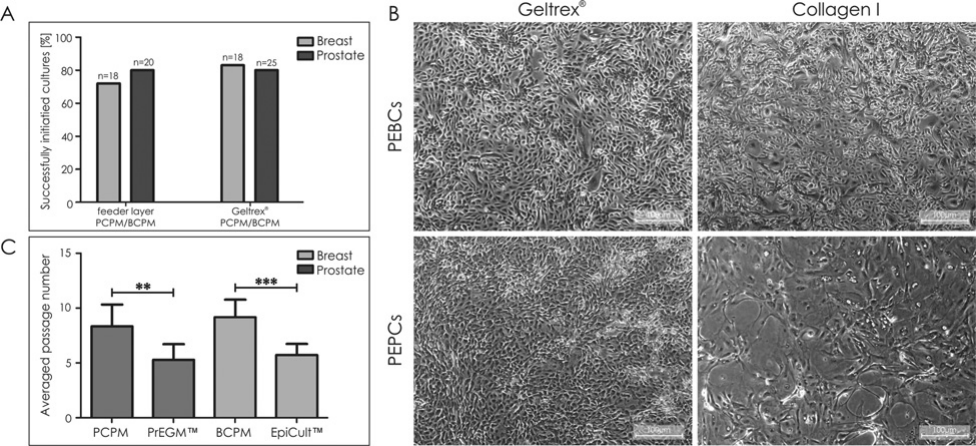
Culturing of PEPCs and PEBCs (**A**) Establishment of primary epithelial cancer cultures in tissue-specific primary cell media on Geltrex® or feeder layer–efficiency comparison. (**B**) Exemplary PEPCs or PEBCs cultured in PCPM/BCPM on Geltrex® or collagen I coating. (**C**) Averaged passage number of PEPCs and PEBCs cultured in tissue-specific and commercial primary cell media. Errors bars indicate SEM. Statistical significance calculated using two-tailed *t*-test; **P  <0.01, ***P  <0.001.

During the experiment course, primary epithelial breast and prostate cancer cells were cultured utilizing protocols differing in terms of media components and plate coatings (Figure 1). Initially, in order to evaluate the efficiency of culturing approaches for culture establishment, both primary epithelial BC and PC cells were cultured in tissue-specific primary cell media–BCPM and PCPM respectively, on Geltrex® and feeder layer plate coatings. Initiation of primary cultures on feeder layer was successful in 80% (16/20) and 72% (13/18) of prostate and breast tumour specimens respectively. Geltrex® coating provided comparable efficiencies of culture initiation in both types of analysed tumour specimens–80% of prostate (20/25) and 83% of breast (15/18) tissue samples initiated primary cultures (Figure 2A). Thus, primary breast and prostate cancer cultures in tissue-specific primary cell media were established with equal efficiency on two different plate coatings–Geltrex® and feeder layer.

### Geltrex® coating is efficient for long-term *in vitro* maintenance of primary epithelial cancer cells

Following establishment on Geltrex®, all successfully initiated primary epithelial prostate cancer cells (PEPCs; 20 cases) were cultured utilizing this coating. Additionally, in order to compare the Geltrex®- and feeder layer-based approaches in terms of culture maintenance efficiency, selected PEPCs established on feeder layer (10 cases) were further cultured on the latter coating. PEPCs were observed to proliferate intensively with comparable passage number reached when using both coatings (*P*  =0.298; Table 2). Selected primary epithelial breast cancer cells (PEBCs; 8 cases) were also subjected to such analysis. Despite no difference was observed in culture initiation efficiency for breast specimens, the number of adherent cells following first two passages was decreasing with each passage. Therefore, an attempt to long-term culture on feeder layer was discontinued in case of BC cells. Nonetheless, all PEBCs established on Geltrex® (15 cases) were successfully maintained in culture using this coating material (Table 2).

**Table 2:** Number of primary epithelial cell passages on different coatings in PCPM/BCPM n/a–not available.

PEPCs				PEBCs		
CASE	Geltrex®	collagen I	feeder layer	CASE	Geltrex®	collagen I
PC17	5	4	6	BC9	9	8
PC21	5	3	5	BC10	5	6
PC24	6	4	8	BC16	6	6
PC32	8	5	11	BC17	6	6
PC35	9	6	8	BC19	9	10
PC38	9	7	10	BC22	12	12
PC39	9	8	n/a	BC27	9	9
PC42	9	4	9	BC32	8	7
PC45	7	5	7	BC46	7	7
PC49	8	6	9	BC49	6	5
PC53	10	6	n/a	BC50	7	7
PC54	8	8	n/a	BC51	9	8
PC55	13	10	11	statistical significance calculated by paired Student's *t*-test		
PC60	7	8	n/a	PEPCs: Geltrex® compared with collagen I–*P* <0.001;		
				Geltrex® compared with feeder layer–*P* =0.298		
PC63	8	5	n/a	PEBCs: Geltrex® compared with collagen I–*P*=0.438		
PC64	6	8	n/a			
PC65	9	7	n/a			
PC66	9	7	n/a			
PC68	7	6	n/a			
PC69	6	8	n/a			

During application of the Geltrex®-based approach no fibroblast expansion was observed, even in higher passages. Impor-tantly, only two passages under such culture conditions enabled to obtain from 5×10^6^ to 1×10^7^ cells capable of further proliferation.

Since Geltrex® is considered quite expensive coating material, we decided to test collagen I as a more cost-effective option. After two initial passages on Geltrex® coating in tissue-specific media, all successfully established cultures (20 PEPCs and 18 PEBCs) were passaged onto collagen I-coated plates. Unfortunately, PEPCs were losing epithelial morphology and reaching lower number of passages (*P*  <0.001; Figure 2B and Table 2). In contrast, collagen I did not have any influence on efficiency of PEBCs culturing (*P*  =0.438; Table 2; Figure 2B).

### Commercial cell media for primary epithelial cancer cells are less effective than tissue-specific primary cell media

Following the second passage, 10 cases of PEPCs and 8 cases of PEBCs were cultured on Geltrex®, concomitantly in PCPM/BCPM or dedicated commercial medium (PrEGM™ or EpiCult™ for prostate and breast cultures respectively). In tissue-specific primary cell media, the cells were able to maintain in culture longer–on an average for three passages (PEPCs–5 compared with 8, *P*  <0.001; PEBCs–6 compared with 9, *P*  <0.001; Figure 2C). Additionally, usage of PCPM/BCPM allowed the cell splitting ratio to double.

**Figure 3:**
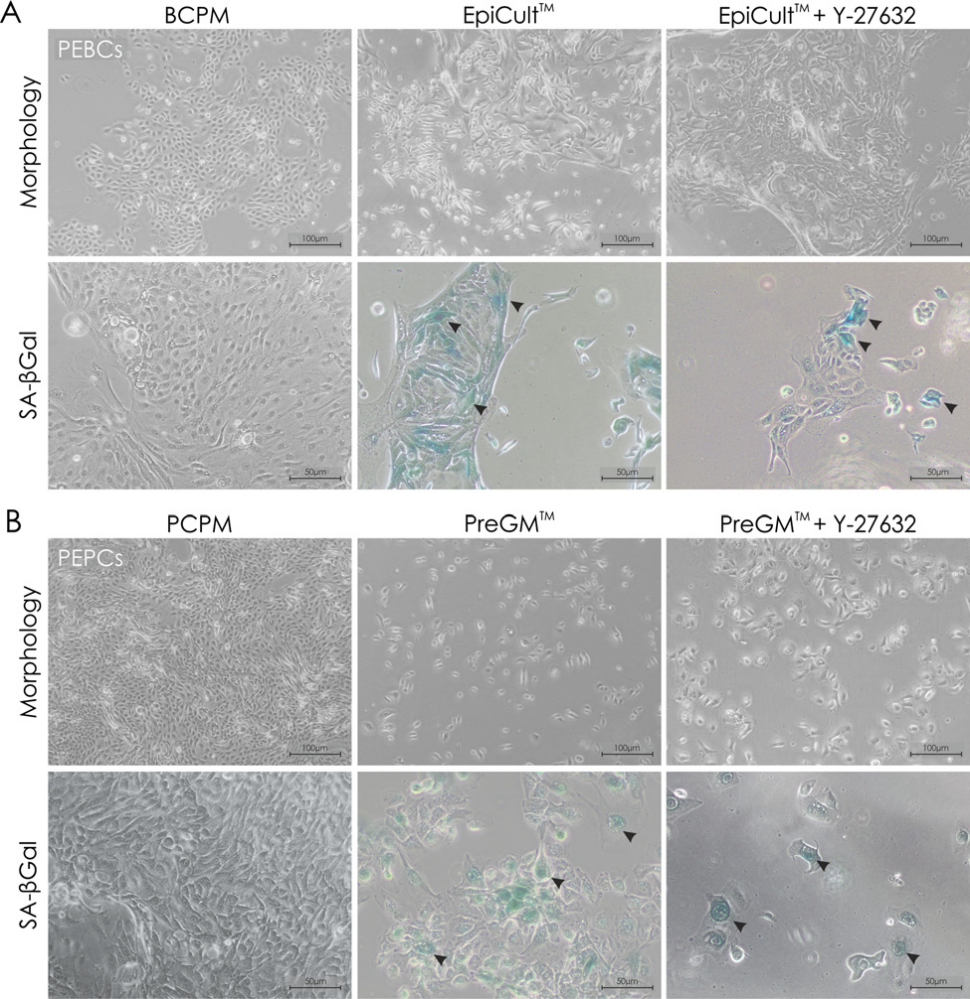
Primary epithelial cancer cells cultured in different media (**A**) Corresponding passages of PEBCs cultured in BCPM, EpiCult™ or EpiCult™ supplemented with ROCK inhibitor (Y-27632) with additional analysis of SA-β-gal activity (blue; arrows mark exemplary SA-β-gal-positive cells). (**B**) Corresponding passages of PEPCs cultured in PCPM, PrEGM™ or PrEGM™ supplemented with ROCK inhibitor (Y-27632), with additional analysis of SA-β-gal activity (blue; arrows mark exemplary SA-β-gal-positive cells).

We hypothesized that ROCK inhibitor (Y-27632) may constitute the key component of primary cancer culture medium. Hence, PEPCs and PEBCs were additionally cultured on Geltrex® in PrEGM™/EpiCult™ supplemented with this inhibitor. Such enrichment, however, did not enhance the effectiveness of culturing approach based on commercial media. In corresponding passages, cells cultured on Geltrex® coating in PCPM/BCPM showed very few visible morphological traits of senescence [increase in size, senescence-associated β-galactosidase (SA-β–gal) activity], on the contrary to cells cultured in commercial medium, even supplemented with ROCK inhibitor (Figure 3).

### Tumour- and tissue-specific markers expression is maintained in PEBCs/PEPCs

**Figure 4:**
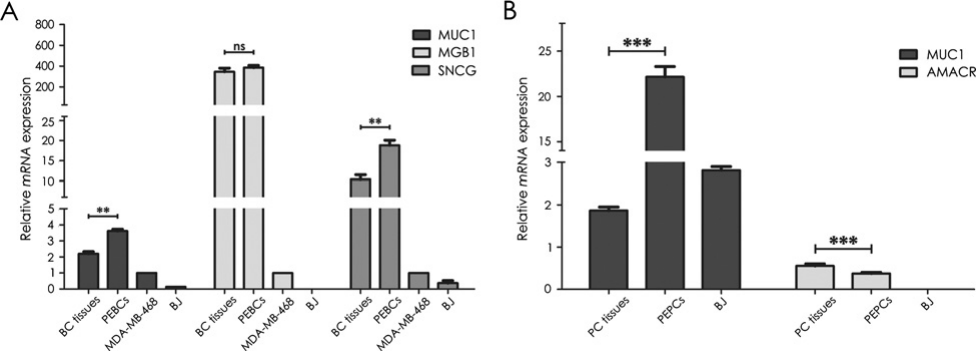
Analysis of specific markers expression at mRNA level (**A**) *MUC1/Y*, *MGB1* and *SNCG* expression in BC tissues compared with PEBCs. (**B**) *MUC1/Y* and *AMACR* expression in PC tissues compared with PEPCs; Analysis was performed for each successfully established sample, averaged expression level is provided. Error bars indicate SEM; **P  <0.01, ***P  <0.001, ns–not statistically significant.

In order to determine whether the primary prostate and breast cultures in fact retained the characteristics of tumour cells, mRNA expression of *MUC1/Y* was measured. Although overexpression of *MUC1* gene is typical for both normal epithelium and epithelial tumour cells, its isoform Y is considered tumour-specific [19,20]. Prostate and breast tumour samples demonstrated low averaged level of this gene expression (1.867±0.082 and 2.200±0.126 respectively; Figure 4) whereas in PEPCs and PEBCs it was ele-vated (22.165 ±1.133 and 3.614±0.108 respectively; Figure 4).

In case of BC, mRNA expression of *MGB1*–marker specific for mammary tissue [21,22] was evaluated and showed comparable averaged levels (*P*  =0.7579) in breast tissue samples and primary cultures (345.945±34.138 and 385.272±21.326 respectively; Figure 4A). On the contrary, *SNCG*–gene associated with BC development [23,24], demonstrated similar pattern as *MUC1/Y*, that is higher level of expression in PEBCs when compared with corresponding tumour samples (18.818±1.245 compared with 10.407±1.089; Figure 4A).

Additional analysis of prostate tumour specimens was focused on expression of *AMACR*–gene encoding an enzyme considered to be overexpressed in PC [25,26], yet with many contradictory data published [27,28], which occurred to be slightly lower in PEPCs than in tissue samples (0.376±0.048 compared with 0.556±0.028; Figure 4B).

**Figure 5:**
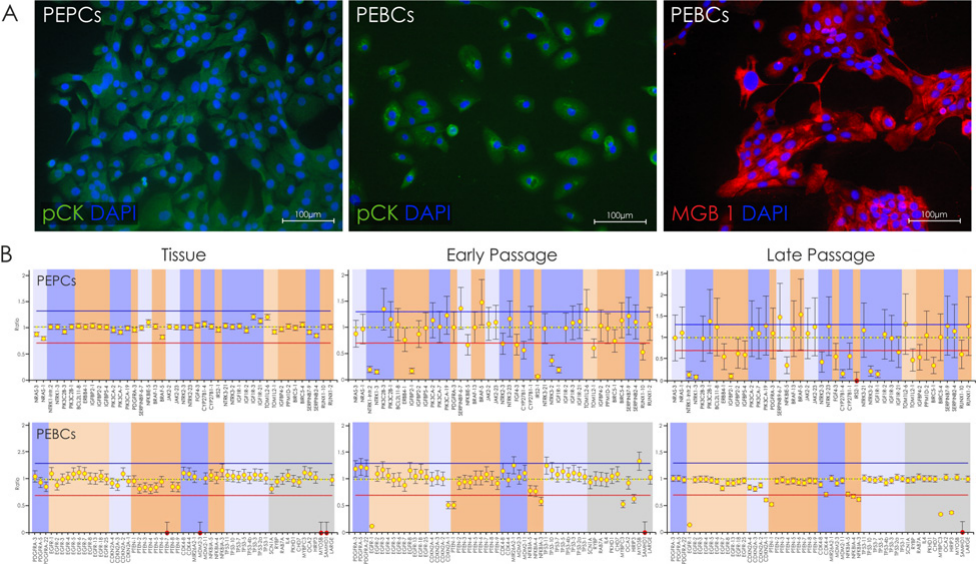
Genetic and phenotypic characteristics of PEPCs and PEBCs (**A**) PEBCs and PEPCs immunostained for characteristic protein markers–cytokeratins (pCK) and mammaglobin A (MBG1). (**B**) MLPA analysis showing tumour-characteristic changes in gene copy number.

To confirm epithelial origin of cells, immunostaining for epithelial markers–cytokeratins, was performed on PEPCs and PEBCs (Figure 5A). Anti-CD90 antibody was employed to visualize any remaining fibroblasts (results not shown). BC cell cultures were additionally analysed for the expression of mammary gland-specific protein, MGB1 (Figure 5A).

### PEPCs and PEBCs maintained original genotype in early and late passages

In order to verify whether tumour-characteristic genomic gains and losses were present in cultures of PEPCs and PEBCs, MLPA method was employed. In case of analysed tissue samples, none or very few alterations were detected (depending on a specimen), most probably due to high percentage of normal cells in surgical samples. Genotype of primary cells was compared between different passages revealing that in both early (<4) and late passages (>4), cancer-specific losses and/or gains were present in PC and BC cells (Figure 5B). Moreover, some new alterations appeared during culture course, probably as an effect of the inevitable molecular dynamics of neoplastic cells (Figure 5B).

### Transduction with immortalizing genes does not extend the PEPCs and PEBCs culture lifespan

In order to extend culture lifespan, primary BC and PC cells were transduced with known immortalizing genes–*SV40LT*, *hEST2* and *BMI-1* (single or in combination). Primary breast and prostate epithelial cells were not susceptible to the effect of applied immortalizing agents, since the approach did not increase the number of passages (Table 3).

**Table 3:** Passage number of primary cancer epithelial cells transduced with immortalizing genes compared with control cells

Primary culture	Immortalizing gene	Number of passages with transduction	Number of passages without transduction
BC22 P:5	*SV40LT*	6	12
	*BMI-1*	6	
	*SV40LT + BMI-1*	4	
	*hEST2*	5	
BC46 P:1	*SV40LT*	5	9
	*BMI-1*	3	
	*SV40LT + BMI-1*	4	
	*hEST2*	5	
BC49 P:1	*SV40LT*	5	9
	*hEST2*	10	
	*SV40LT + hEST2*	10	
BC50 P:1	*SV40LT*	9	8
	*hEST2*	8	
	*SV40LT + hEST2*	3	
BC51 P:1	*SV40LT*	3	8
	*hEST2*	8	
	*SV40LT + hEST2*	3	
PC21 P:4	*SV40LT*	7	7
PC38 P:1	*SV40LT*	7	8

## DISCUSSION

Despite the complexity of their establishment and maintenance, primary cancer cultures begin to be considered the most adequate platform for *in vitro* analytical purposes. In the present study, we provide a simplified approach to primary breast and prostate epithelial cancer cells culture, utilizing extracellular matrix reconstitution and tissue-specific primary cell medium.

Establishment of PEPCs and PEBCs under these conditions demonstrated equal efficiency to culture initiation on feeder layer. The main advantage of Geltrex®-based approach is simple plate preparation (e.g. less complicated and time-consuming coating procedure) and standardization (e.g. no need to seed the specified number of fibroblasts). Feeder layer is associated with numerous technical issues, including incomplete inactivation of fibroblasts as well as their tendency to lose contact inhibition, transform or even become unresponsive to mitomycin C in a long-term culture [16]. Therefore, there is a risk that feeder cells will not be able to provide epithelial cancer cells with adequate support or even contaminate the culture. Surprisingly, comparison of Geltrex® and feeder layer coating in terms of culture maintenance also gave similar outcomes, contrary to the previous suggestions on almost unlimited proliferation of epithelial cells provided by feeder layer approach [15]. Moreover, when PEPCs or PEBCs were cultured on Geltrex® coating but in commercial primary cell dedicated media, the culturing efficiency was noticeably lower, even following supplementation with ROCK inhibitor. Initially, ROCK inhibitor was applied in cultures of stem cells to enable more efficient culturing of dissociated spheres [29] and keratinocytes to enhance their proliferation and culture time [30]. Afterwards, this inhibitor was reported to constitute a key factor in long-term culture maintenance of normal and neoplastic cells from various tissues of origin [15].

Change of plate coating to collagen I negatively influenced phenotype and proliferation of PEPCs but not PEBCs. Such a difference is consistent with current availability of stable cancer cell lines derived from these tumour types–there is a plenty of BC cell lines, whereas the selection of possible testing platform for PC is severely limited (lack of stable cancer cell line from the primary tumour site [6,7]). Each of the very few commercially available PC lines was established from metastatic sites (mostly bone metastases) or tumour xenografts (usually implanted heterotopically). Such issue may not only indicate that this tumour type is troublesome and more demanding under *in vitro* conditions but also emphasize the need for more suitable PC model, for which the primary cells constitute a possible advantageous option.

Importantly, cells cultured on Geltrex® coated plates in tissue-specific primary cell medium were maintaining their tumour and tissue-specific characteristics. In case of tumour specific markers–*MUC1/Y* and *SNCG*, mRNA expression was elevated in culture, suggesting that analysed tissue samples were constituting heterogeneous populations, with high percentage of normal, non-neoplastic cells, which were negatively selected during culture course. *MGB1* expression was detected at similar level in both BC specimens and PEBCs, which was in line with our expectations, since this marker is specific for mammary gland, both normal and neoplastic tissue [21,22]. On the contrary, there are very few markers that may be considered PC specific. Latest literature reports indicate the important role of *AMACR* gene, however, it is not clear whether it can be considered tissue or cancer specific marker [25–28]. Our analysis demonstrated that in contrary to other analysed markers, the expression of *AMACR* observed in tissue samples decreased during culture course (Figure 4B). Considering the fact that role of this gene in PC is not fully understood, it is not clear why pattern of its expression differs from other analysed markers. Additionally, it may be concluded on the example of *MUC1/Y* expression analysis that the percentage of normal cells in tissue specimens was higher in prostate than in breast samples (greater difference in *MUC1/Y* expression between tissues and corresponding cultures).

Interestingly, when cultured in dedicated commercial media, primary epithelial cancer cells tend to be characterized by earlier onset of senescence than those maintained in tissue-specific primary cell media. Despite the fact that unlimited proliferation potential is commonly considered as one of the most characteristic features of cancer cells [31], primary cancer cells are known to undergo *in vitro* senescence [10,11]. This phenomenon is inseparably associated with alterations in the molecular context of the cell [32,33], especially factors implicated in cell-cycle regulation [34]. Such a phenomenon, however, is also observed in stable cancer cell lines. Consequently, we have recently reported that even population of cancer cells with almost infinite proliferative potential is characterized by low percentage of senescent cells [11].

Generally, stable cell lines either stabilize due to spontaneous immortalization (very rare event, mostly due to acquisition of new mutations) or are obtained *via* isolation from xenografts or treatment with immortalizing agents (of viral, chemical or other origin). Obviously, the last approach is associated with the risk of altering molecular profile of the cell and hindering the precise reflection of *in vivo* state [35,36]. Nevertheless, as our approach did not prevent the inevitable inhibition of *in vitro* cancer cells proliferation, we made an attempt to transduce primary cancer cells with well-known immortalizing genes–*SV40LT* [37], *BMI-1* [38] and *hEST2* [39]. None of the utilized genes significantly prolonged culture time, neither in breast nor in prostate cells. It is worth mentioning that primary cancer cells are less prone to genetic manipulations than normal cells. Scientific reports provide several methods for normal cells immortalization, based on induction of various genes expression, e.g. *c-Myc* [40,41] or *hTERT* combined with *E7* or the *SV40LT* [42]. On the contrary, only few successful attempts to immortalize primary cancer cells were described in the literature, including induction of *hTERT* [43] or *E6* and *E7* genes of *HPV-16* [42]. Generally, it may be concluded that difference in susceptibility of primary cancer cells to ‘immortalization’ results from the molecular context of cancer cells.

Stable cell lines, the most common *in vitro* model for cancer studies, are successfully derived only from a small fraction of tumours, usually those highly aggressive. Undoubtedly, such platforms do not provide the representation of the whole spectrum of tumour subpopulations. Therefore, primary cell cultures, reflecting high heterogeneity of cells within a tumour, seem to be an essential tool for reliable studies on cancer treatment, drug development and cancer biology. Proposed protocol is an efficient way to obtain primary epithelial cancer cells, retaining their molecular characteristics and providing *in vitro* expansion sufficient for analytical purposes. As primary cancer cells are gaining recognition among researchers in all oncology-related fields, a standardized approach for their *in vitro* maintenance is of considerable value. Importantly, availability of primary cancer cells derived from a particular patient may facilitate the attempts to personalize anticancer therapy. In the era of molecular diagnostics, testing drugs or their combinations directly on patient tumour material seems a powerful approach towards improvement of anticancer treatment efficiency.

## Abbreviations

AMACR: α-methylacyl-CoA racemase
BC: breast cancer
BCPM: breast cancer primary medium
HBSS: Hank's balanced salt solution
*MGB1*: mammaglobin 1
MLPA: multiplex ligation-dependent probe amplification
PC: prostate cancer
PCPM: prostate cancer primary medium
PEBC: primary epithelial breast cancer cell
PEPC: primary epithelial prostate cancer cell
ROCK: Rho kinase
SA-β-gal: senescence-associated β-galactosidase
*SNCG*: synuclein gamma
